# Saponins of ginseng products: a review of their transformation in processing

**DOI:** 10.3389/fphar.2023.1177819

**Published:** 2023-04-28

**Authors:** Xian-Wen Ye, Chun-Shuai Li, Hai-Xia Zhang, Qian Li, Shui-Qing Cheng, Jia Wen, Xuan Wang, Hong-Min Ren, Liang-Jing Xia, Xu-Xing Wang, Xin-Fang Xu, Xiang-Ri Li

**Affiliations:** ^1^ Centre of TCM Processing Research, Beijing University of Chinese Medicine, Beijing, China; ^2^ Beijing Key Laboratory for Quality Evaluation of Chinese Materia Medica, Beijing University of Chinese Medicine, Beijing, China; ^3^ Institute of Regulatory Science for Traditional Chinese Medicine, Beijing University of Chinese Medicine, Beijing, China

**Keywords:** red ginseng products, ginsenosides, transformation rule, herbal medicine, pharmacological activities

## Abstract

The primary processed product of *Panax ginseng* C.A. Meyer (*P. ginseng*) is red ginseng. As technology advances, new products of red ginseng have arisen. Red ginseng products, e.g., traditional red ginseng, sun ginseng, black ginseng, fermented red ginseng, and puffed red ginseng, are commonly used in herbal medicine. Ginsenosides are the major secondary metabolites of *P. ginseng*. The constituents of *P. ginseng* are significantly changed during processing, and several pharmacological activities of red ginseng products are dramatically increased compared to white ginseng. In this paper, we aimed to review the ginsenosides and pharmacological activities of various red ginseng products, the transformation law of ginsenosides in processing, and some clinical trials of red ginseng products. This article will help to highlight the diverse pharmacological properties of red ginseng products and aid in the future development of red ginseng industrialization.

## 1 Introduction

*Panax ginseng* C.A. Meyer (*Panax ginseng*) is an ancient Chinese medicinal material used in Asian nations for over 2,000 years (Kennedy and Scholey, 2003). It has been listed as a medicinal herb in *Shennong Bencao Jing*, a standard Chinese herbal dictionary. *P. ginseng*, *Panax quinquefolius* L., and *P. notoginseng* (Burk) F.H. Chen (*P. notoginseng*), all from the Araliaceae family, are widely used herbs. Several studies over the past few decades have shown that *P. ginseng* and *P. notoginseng* have various pharmacological effects on immunological and neurological system disorders (Liu et al., 2020). The kinds and concentrations of their primary active ingredients, saponins, may be altered throughout the steaming process, and the therapeutic efficacies of raw and steaming *P. ginseng* and *P. notoginseng* vary. These variations in saponins are causally significant (Zhang et al., 2019). Meanwhile, according to traditional Chinese medicine (TCM) theory, their uses differ, since *P. ginseng* strengthens vital energy while *P. notoginseng* encourages blood circulation (Xiong et al., 2022).

*P. ginseng* is often processed into white ginseng (WG) and traditional red ginseng (TRG), most well-used in clinical applications for their great pharmacological activity. In TCM, the steaming method of *P. ginseng* is initially listed in the Complete Manual of Experience in the Treatment of Sores. The character of TRG is detailed and described in The Ming dynasty’s Enlightening Primer of Materia Medica. In steaming processing, the quality of TRG improves with an increase of *P. ginseng* cultivation age. Typically, TRG is produced by six-year-old ginseng in Korea. In addition, the unique and advanced processing technology makes Korean red ginseng (KRG) predominate the world ginseng market. With the development of steaming, fermenting, and puffing technology, many new red ginseng products are being produced, including sun ginseng (SG), black ginseng (BG), fermented red ginseng (FRG), and puffed red ginseng (PRG). These process conditions directly influence the pharmacological activity of red ginseng.

It is widely known ginsenosides can be classified into three types: the protopanaxadiol type (Rb1, Rc, Rb2, and Rd); the protopanaxatriol type (ginsenoside Rg1 and Re); and the oleanolic acid type (ginsenoside Ro and polyacetylene ginsenoside Ro) according to different aglycones (Xu et al., 2014). The various structures of ginsenosides endow them with rich pharmacological activities, such as antioxidation, anti-inflammation, anti-apoptosis, and so on (Choi et al., 2014; Li et al., 2014). Additionally, ginsenosides can be divided into major and rare ginsenosides according to the different content of ginseng, which all have significant pharmacological activities (Wei et al., 2011).

The major ginsenosides occur in WG, TRG, and other new types of red ginseng. In contrast, rare ginsenosides are present in TRG at a trim level, including Rg2, Rg3, Rh1, and Rh2. However, compared with TRG, the rare ginsenosides are abundant in the new types of red ginseng. The types and contents of ginsenosides in different red ginseng products result in various pharmacological activities, and the relationship between ginsenosides and their bioactivities help in the application of red ginseng products in clinical settings.

We aimed to review the relevant clinical studies of red ginseng products and summarize the discovered ginsenoside components of red ginseng. Furthermore, we discuss the structure–functional relationship of ginsenosides. The transformation law of ginsenosides in red ginseng processing are revealed to illustrate the medicinal composition transformation. The other pharmacological activities of red ginseng, such as TRG, SG, BG, FRG, and PRG, are also discussed. We summarize the process and character of the new type of red ginseng, which provides the basis for further research to facilitate the development of red ginseng industrialization in the future.

## 2 Red ginseng products

### 2.1 TRG

Generally, TRG is steamed at 90°C–100°C for 2-3 h and then dried (Chung et al., 2014). The large-scale application of TRG began in the Qing Dynasty. According to TCM theory, WG is used to “supply qi and promote the production of body fluids” and enhance physical fitness and disease resistance. In contrast, TRG is often used to “boost yang” and replenishing vital essence with the “warming effect” (Zhang et al., 2012).

**FIGURE 1:**
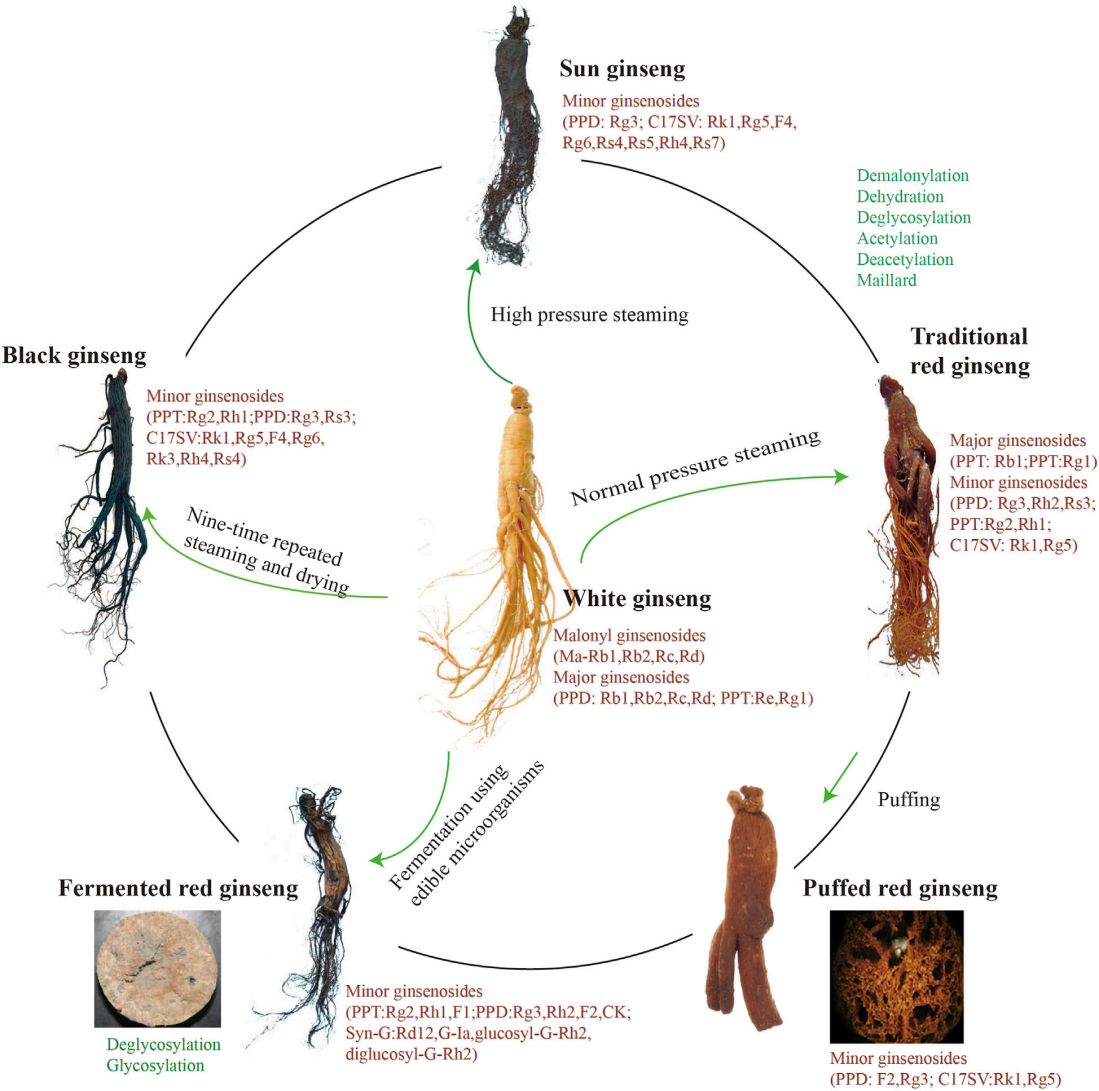
Various types of red ginseng.

During TRG processing, one ginsenoside can be transformed into another by demalonylating, decarboxylating, deglycosylating, and dehydrating (Figure 1). Compared with WG, the rare ginsenosides are the characteristic compound with significant pharmacological activities. TRG exhibits more potent anticancer activity than WG due to the abundance of rare ginsenosides generated from processing (Li et al., 2011; Kim J. H. et al., 2014), which has been further developed into drugs and health products. The pharmacological activity of TRG focuses on anti-aging (Peng et al., 2021), treating erectile dysfunction (Jang et al., 2008), immune-modulating (Kim et al., 2021), the antidepressant effect (Lee et al., 2020), and anti-inflammation (Min et al., 2022). In addition, TRG can inhibit tau aggregation and promote tau dissociation *in vitro*, which can be a potential therapeutic agent to treat neurodegenerative diseases (Shin et al., 2020).

### 2.2 SG

SG is prepared by steaming fresh ginseng at a temperature of 120°C or higher, which is a higher temperature than during TRG processing (Figure 1). SG contains approximately equal amounts of three major ginsenosides, Rg3, Rg5, and Rk1, in a higher concentration than TRG. One study (Kim et al., 2000) first reported this processed red ginseng, which is more potent in its ability to induce endothelium-dependent relaxation and free radical scavenging activity.

Compared with TRG, SG contains an abundance of ginsenosides Rg3, Rg5, and Rk1 (Kwon et al., 2001). In addition, new acetylated ginsenosides (Rs4, Rs5, Rs6, and Rs7) and dammarane glycosides (Rk1, Rk2, and Rk3) have been isolated from SG (Park et al., 2002a; Park et al., 2002b). These different types and amounts of ginsenosides endow SG with more potent pharmacological effects than TRG under certain pathological conditions. SG reportedly serves several functions, including free radical scavenging (Kang et al., 2006), peroxynitrite scavenging (Kang et al., 2009), antitumor-promoting (Song et al., 2012), antihyperglycemic (Jiao et al., 2014), and cytoprotection activities (Lee C. S. et al., 2012). Moreover, SG has memory-enhancing activities (Lee C. H. et al., 2013), and enhances cognitive function in patients with moderately severe Alzheimer’s disease (Heo et al., 2012).

### 2.3 BG

Due to repeated steaming and drying processes, BG is named according to its surface color change (Figure 1). The nine-time repeated steaming and drying is a typical method in TCM, mainly to rectify the properties and increase the components of Chinese medicine. Observations indicate that BG improves at temperatures of 95°C or higher, at least from the appearance (Oh et al., 2021). Multiple steaming and drying can enhance the antibacterial activity of BG (Lee H. W. et al., 2021). Compared with the TRG, the total saponins of BG are absorbed faster in the gut and exposed more widely (Yoo et al., 2021). In recent years, this method has been used to manufacture functional food.

After nine repeated steaming cycles, ginseng gradually becomes BG, with increased Rg3 content (Kim, 2015). Nineteen ginsenosides (Rg1, Re, Rf, Rb1, Rc, Rb2, Rd, F4, Rg6, Rk3, Rh4, 20(*S*)-, 20(*R*)-Rg3, 20(*S*)-, 20(*R*)-Rs3, Rk1, Rg5, Rs4, and Rs5) have been determined to be found in BG; among them, the ginsenosides Rg3, Rg5, and Rk1 are the main components (Sun et al., 2009). Research has also revealed that the total ginsenosides increase with number of steam cycles (Lee H. et al., 2012), and that the fructose in BG is 44 times that present in WG and 18.3 times that in RG (Zhu et al., 2019).

In animal and cell culture models, BG plays a role in the prevention and treatment of diseases such as cognitive impairment (Park et al., 2011), obesity (Park et al., 2019), fetal alcohol syndrome (Lee et al., 2009), and breast cancer (Kim and Kim, 2015). BG is a potential anti-aging supplementation, which can reduce the activation of p53-dependent p21 and p16 classical aging pathways in the liver, skeletal muscle, and white fat (Lee S. et al., 2022). BG can prevent liver injury by resisting oxidative stress, regulating lipid and glucose metabolism, and reducing inflammation and TLR4/NF-κB axis (Jiang et al., 2021; Wei et al., 2022). BG may maintain endothelial integrity by activating Akt, reducing vascular protein leakage, leukocyte infiltration, and proinflammatory factor release in alveolar lavage fluid, and thus plays a protective role against particle-induced lung injury and vascular hyperpermeability (Lee et al., 2019; Kim et al., 2022).

### 2.4 FRG

Fermenting food through edible microorganisms can produce other active small molecular compounds, which has aroused the interest of most food scientists and dietitians (Oh et al., 2015). Similarly, the fermentation applied in the red ginseng process, which is called FRG, produces a significant change in ginsenoside derivatives and possesses great pharmacological activity (Irfan et al., 2022), such as anti-inflammatory (Kim et al., 2018; Bae et al., 2021), antioxidant (Saba et al., 2018), anti-allergy (Kim et al., 2019), anti-diabetes (Jang et al., 2017), anti-anxiety (Han et al., 2020), and so on. There is another FRG, which involves ginseng steaming, extracting, fermenting, and freeze-drying. The pharmacodynamic function of ginseng after fermentation is improved, which may be related to the increase in ingestion rates and absorption levels of *P. ginseng* (Lee et al., 2015).

Bacteria and fungi, such as *Lactobacillus*, *Bifidobacterium*, *Saccharomyces cerevisiae*, and Red-Koji, mainly perform the fermentation. These microbes are essential in transforming ginseng components (Lee, et al., 2015; Choi et al., 2016). Moreover, mushroom mycelia can provide the microbial environment needed in ginseng fermentation (Bae et al., 2011). Interestingly, fermentation creates a new small molecule, Compound K (CK). CK is transformed from Rb1, Rb2, Rc, and Rd, the metabolite of FRG digested by intestinal microorganisms, and has considerable activity against diabetes, cancer, and immune stimulation (Jung et al., 2019; Kim et al., 2020). In addition, a large amount of Rg3 is fermented into Rh2 by lactic acid bacteria, which have obvious anti-tumor, anti-allergic, and anti-inflammatory activities (Bae et al., 2004; Trinh et al., 2007).

Purple-red ginseng is prepared by fresh ginseng and then through fermentation, steam ripening, and low-temperature ripening. This processing uses bilberry, sugar, and bulgaricus. The ginsenoside, Rg5, is the most abundant ingredient of this FRG. In addition, this FRG might be a beneficial therapeutic supplementary substitute for metabolic syndrome with the efficacy to improve insulin sensitivity and lower postprandial glucose levels (Kho et al., 2016; Lee S. J. et al., 2022).

### 2.5 PRG

After puffing, the texture of ginseng is loose and porous, and easy to dehydrate. The process goes through rapid heating at atmospheric pressure and immediate pressure reduction, and the product is what we often call PRG (Hoseney, 1986).

The red ginseng was puffed with rice to avoid a burning phenomenon at high temperatures (An et al., 2011). PRG shows higher extraction yield and crude saponin content than non-puffed ginseng (Kim et al., 2008). The primary ginsenosides (Rb1, Rb2, Rc, Rd, Re, and Rg1) are effectively converted into minor ginsenosides (F2, Rg3, Rk1, and Rg5) by puffing, which is similar to the effect of the steaming process on the content of transformed ginsenosides. However, the time required for the transformation of ginsenosides by steaming (4–36 days) is much longer than by puffing (less than 30 min) (Shin et al., 2019). During *in vitro* experiments, the ethanolic extract of PRG shows a more vital antioxidant capacity than TRG. In addition, more robust antioxidant properties are observed in bulk oil and oil-water emulsions (Lee et al., 2018).

## 3 Ginsenosides in red ginseng and transformation in processing

### 3.1 Ginsenosides in red ginseng

**FIGURE 2:**
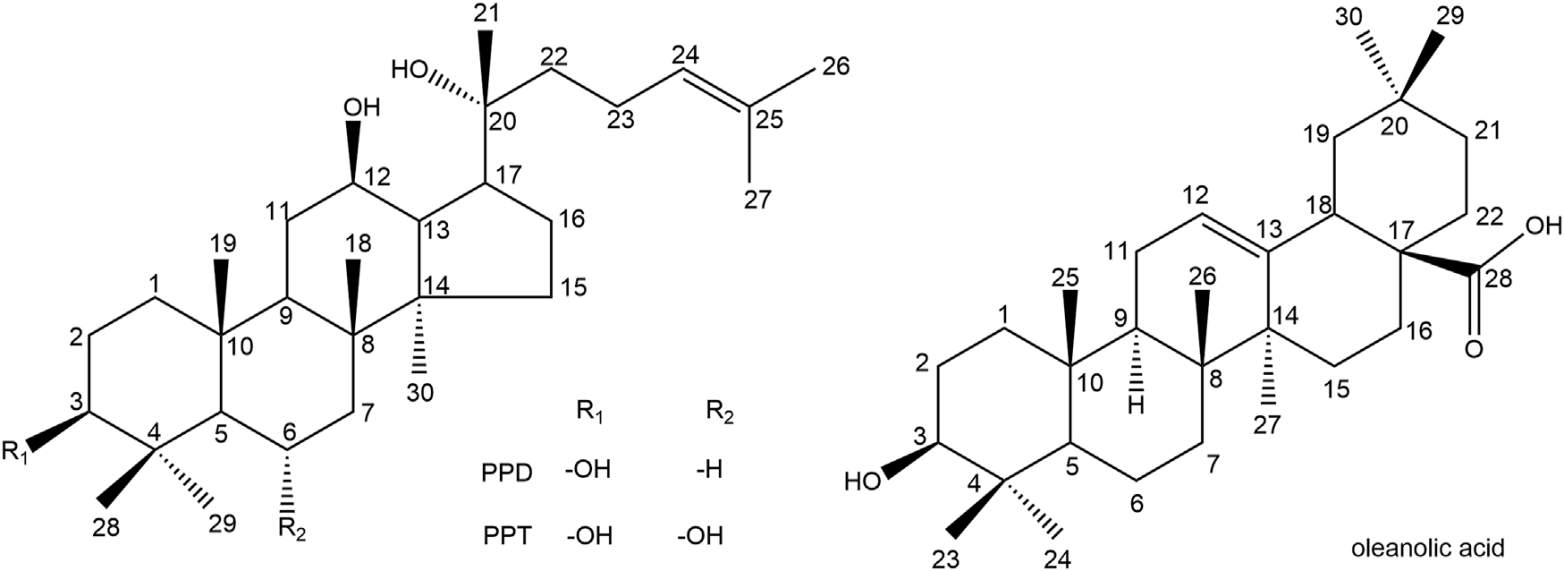
Structure of protopanaxadiol (PPD), protopanaxatriol (PPT), and oleanolic acid (OA).

Ginsenosides are the main active components in red ginseng. So far, about 100 ginsenosides have been discovered in red ginseng (Table 1), and the number is still increasing. In red ginseng products, protopanaxadiol (PPD), protopanaxatriol (PPT), and oleanane (OLE) ginsenosides are the main classification of ginsenosides (Figure 2). Ginsenoside is a triterpenoid saponin with a dammarane skeleton. The ether bond combines carbon-3, carbon-6, or carbon-20 with glycosyl residue. Carbon-20 is a chiral carbon atom presenting the 20(*S*) and 20(*R*) epimers, such as ginsenoside Rg3, Rh2, Rs3, Rg2, and Rh1. Furthermore, dehydration in the carbon-20 can present positional isomers of the double bond at carbon-20 (21) or carbon-20 (22), while the double bond at carbon-20 (22) also presents cis-trans isomers, such as Rg9, F4, Rs6, Rh4, Rs4, Rg5, and Rh3.

**TABLE 1:** Ginsenosides in red ginseng products.

No.	Name	Formula	Plant material	References
1	Acetyl-20(*S*)-ginsenoside Ra1	C_60_H_100_O_27_	TRG	Xu et al. (2016)
2	Acetyl-20(*S*)-ginsenoside Ra2	C_60_H_100_O_27_	TRG	Xu et al. (2016)
3	20(*S*)-ginsenoside Ra1	C_58_H_98_O_26_	TRG	Kasai et al. (1983)
4	20(*S*)-ginsenoside Ra2	C_58_H_98_O_26_	TRG	Kasai et al. (1983), Zhou et al. (2016)
5	20(*S*)-ginsenoside Ra3	C_59_H_100_O_27_	TRG	Zhou et al. (2016)
6	20(*S*)-ginsenoside Rb1	C_54_H_92_O_23_	TRG, BG, FRG, PRG	Xu et al. (2016), Zhou et al. (2016)
7	20(*S*)-ginsenoside Rb2	C_53_H_90_O_22_	TRG, BG, FRG, PRG	Xu et al. (2016), Zhou et al. (2016)
8	20(*S*)-ginsenoside Rb3	C_53_H_90_O_22_	TRG	Xu et al. (2016), Zhou et al. (2016)
9	20(*S*)-ginsenoside Rc	C_53_H_90_O_22_	TRG, BG, FRG, PRG	Xu et al. (2016), Zhou et al. (2016)
10	20(*S*)-ginsenoside Rd	C_48_H_82_O_18_	TRG, BG, FRG, PRG	Xu et al. (2016), Zhou et al. (2016)
11	20(*S*)-ginsenoside Rg3	C_42_H_72_O_13_	TRG, SG, BG, FRG, PRG	Zhou et al. (2016)
12	20(*R*)-ginsenoside Rg3	C_42_H_72_O_13_	TRG, SG, BG, FRG,PRG	Zhou et al. (2016)
13	20(*R*)-ginsenoside Rh2	C_36_H_62_O_8_	TRG, FRG	Kitagawa et al. (1983)
14	20(*S*)-ginsenoside Rh2	C_36_H_62_O_8_	TRG, FRG	Kitagawa et al. (1983)
15	20(*S*)-ginsenoside Rs1	C_55_H_92_O_23_	TRG	Xu et al. (2016), Zhou et al. (2016)
16	20(*S*)-ginsenoside Rs2	C_55_H_92_O_23_	TRG	Xu et al. (2016), Zhou et al. (2016)
17	20(*S*)-ginsenoside Rs3	C_44_H_74_O_14_	TRG, BG	Baek et al. (1997), Zhou et al. (2016)
18	20(*R*)-ginsenoside Rs3	C_44_H_74_O_14_	TRG, BG	Zhou et al. (2016)
19	Malonyl-20(*S*)-ginsenoside Rb1	C_57_H_94_O_26_	TRG	Xu et al. (2016)
20	Malonyl-20(*S*)-ginsenoside Rb2	C_56_H_92_O_25_	TRG	Xu et al. (2016)
21	Malonyl-20(*S*)-ginsenoside Rd	C_51_H_84_O_21_	TRG	Xu et al. (2016)
22	20(*S*)-notoginsenoside R4	C_59_H_100_O_27_	TRG	Xu et al. (2016)
23	20(*S*)-pseudo-ginsenoside RC1	C_50_H_84_O_19_	TRG	Xu et al. (2016)
24	20(*S*)-quinquenoside R1	C_56_H_94_O_24_	TRG	Xu et al. (2016), Zhou et al. (2016)
25	20(*S*)-ginsenoside F2	C_42_H_72_O_13_	PRG	An et al. (2011)
26	20(*S*)-ginsenoside Compond-K	C_36_H_62_O_8_	FRG	Hasegawa (2004)
27	20(*S*)-notoginsenoside Fa	C_59_H_100_O_27_	TRG	Xu et al. (2016)
28	20(*S*)-notoginsenoside Q	C_63_H_106_O_30_	TRG	Xu et al. (2016)
29	20(*S*)-notoginsenoside Ra3	C_59_H_100_O_27_	TRG	Xu et al. (2016)
30	20(*S*)-notoginsenoside Ra1	C_58_H_98_O_26_	TRG	Xu et al. (2016)
31	20(*S*)-notoginsenoside Ra2	C_58_H_98_O_26_	TRG	Xu et al. (2016)
32	20(*S*)-notoginsenoside Fc	C_58_H_98_O_26_	TRG	Xu et al. (2016)
33	20(*S*)-quinquenoside III	C_51_H_86_O_21_	TRG	Xu et al. (2016)
34	20(*S*)-ginsenoside Re	C_48_H_82_O_18_	TRG, BG, PRG	Xu et al. (2016), Zhou et al. (2016)
35	Acetyl-20(*S*)-ginsenoside Re	C_50_H_84_O_19_	TRG	Xie et al. (2012)
36	20(*S*)-ginsenoside Re2	C_48_H_82_O_19_	TRG	Zhou et al. (2016)
37	Acetyl-20(*S*)-ginsenoside Rf	C_44_H_74_O_15_	TRG	Xu et al. (2016)
38	20(*S*)-ginsenoside Rf	C_42_H_72_O_14_	TRG, BG	Zhou et al. (2016)
39	20(*R*)-ginsenoside Rf	C_42_H_72_O_14_	TRG	Lee et al. (2013a), Zhou et al. (2016)
40	20(*S*)-ginsenoside Rf-1a	C_42_H_72_O_14_	TRG	Zhou and Yang (2015), Zhou et al. (2016)
41	Acetyl-20(*S*)-ginsenoside Rg1	C_44_H_74_O_15_	TRG	Xu et al. (2016)
42	20(*S*)-ginsenoside Rg1	C_42_H_72_O_14_	TRG, BG, PRG	Zhou et al. (2016)
43	20(*S*)-ginsenoside Rg2	C_42_H_72_O_13_	TRG	Zhou et al. (2016)
44	20(*R*)-ginsenoside Rg2	C_42_H_72_O_13_	TRG	Zhou et al. (2016)
45	20-gluco-20(*S*)-ginsenoside Rf	C_48_H_82_O_19_	TRG	Zhou et al. (2016)
46	20(*S*)-ginsenoside Rh1	C_36_H_62_O_9_	TRG	Xu et al. (2016), Zhou et al. (2016)
47	20(*R*)-ginsenoside Rh1	C_36_H_62_O_9_	TRG	Xu et al. (2016), Zhou et al. (2016)
48	20(*S*)-notoginsenoside R1	C_47_H_80_O_18_	TRG	Xu et al. (2016), Zhou et al. (2016)
49	20(*S*)-notoginsenoside R2	C_44_H_74_O_15_	TRG	Zhou et al. (2016)
50	20(*R*)-notoginsenoside R2	C_44_H_74_O_15_	TRG	Zhou et al. (2016)
51	3β,12β-dihydroxydammar-20 (22) E, 24-diene-6-O-β-D-xylopyranosyl-(1→2)-O-β-D-glucopyranoside	C_41_H_68_O_12_	TRG	Zhou et al. (2016)
52	12-O-glucoginsenoside Rh4	C_42_H_70_O_13_	SG	Cho et al. (2013)
53	20 (22) Z-ginsenoside Rh4	C_36_H_60_O_8_	TRG, SG, BG	Zhou et al. (2016)
	20 (22) E-ginsenoside Rh4	C_36_H_60_O_8_	TRG, SG, BG	Baek et al. (1996), Zhou et al. (2016)
54	12β,25-dihydroxydammar-20 (22) E-ene-3-O-β-D-glucopyranosyl-(1→2)-O-β-D-glucopyranoside	C_42_H_72_O_14_	TRG	Zhou et al. (2016)
55	20 (22) E-ginsenoside Rg11	C_42_H_70_O_15_	SG	Cho et al. (2013)
56	23-O-methylginsenoside Rg11	C_43_H_72_O_15_	TRG	Zhou and Yang, (2015); Zhou et al. (2016)
57	20 (22) E-ginsenoside Rh10	C_36_H_62_O_9_	SG	Cho et al. (2013)
58	20 (22) E-ginsenoside F4	C_42_H_70_O_12_	TRG, BG	Ryu et al. (1996), Zhou et al. (2016)
59	20 (22) Z-ginsenoside F4	C_42_H_70_O_12_	TRG, BG	Zhou et al. (2016)
60	Ginsenoside Rf2	C_42_H_70_O_14_	TRG	Park et al. (1998)
61	Ginsenoside Rg5	C_42_H_70_O_12_	TRG, SG, BG, FRG, PRG	Xu et al. (2016), Zhou et al. (2016)
62	Ginsenoside Rz1	C_42_H_70_O_12_	TRG, SG	Zhou et al. (2016)
63	Ginsenoside Rg6	C_42_H_70_O_12_	TRG, BG	Zhou et al. (2016)
64	Ginsenoside Rk1	C_42_H_70_O_12_	SG, TRG, BG, PRG	Zhou et al. (2016)
65	Ginsenoside Rk2	C_42_H_60_O_7_	SG	Park et al. (2002a)
66	Ginsenoside Rk3	C_42_H_60_O_8_	SG, TRG, BG	Park et al. (2002b); Zhou et al. (2016)
67	20 (22) E-ginsenoside Rs4	C_44_H_72_O_13_	SG, BG	Park et al. (2002a)
68	20 (22) Z-ginsenoside Rs4	C_44_H_72_O_13_	TRG, SG, BG	Zhou and Yang, (2015); Zhou et al. (2016)
69	Ginsenoside Rs5	C_44_H_72_O_13_	SG, BG	Park et al. (2002b)
70	Ginsenoside Rs6	C_38_H_62_O_9_	SG	Park et al. (2002a)
71	Ginsenoside Rs7	C_38_H_62_O_9_	SG	Park et al. (2002b)
72	20 (22) Z-ginsenside Rg9	C_42_H_70_O_13_	TRG	Lee et al. (2013b)
73	20 (22) E-ginsenside Rg9	C_42_H_70_O_13_	TRG	Lee et al. (2013a); Zhou et al. (2016)
74	Ginsenside Rg10 (8)	C_42_H_70_O_13_	TRG	Lee et al. (2013b)
75	Ginsenoside Rg4	C_42_H_70_O_12_	TRG	Xu et al. (2018)
76	20 (22) Z-ginsenoside Rh3	C_36_H_60_O_7_	TRG	Kim et al. (1996)
77	Ginsenoside Ro	C_48_H_76_O_19_	TRG	Zhou et al. (2016)
78	Ginsenoside Ro methyl ester	C_49_H_78_O_19_	TRG	Zhou et al. (2016)
79	Polyacetyleneginsenoside Ro	C_65_H_100_O_21_	TRG	Zhou et al. (2016)
80	Ginsenoside-Ro-6′-butyl ester	C_52_H_84_O_19_	TRG	Zhou and Yang (2015); Zhou et al. (2016)
81	Chikusetsusaponin IVa methyl ester	C_43_H_68_O_14_	TRG	Zhou et al. (2016)
82	Chikusetsusaponin IVa butyl ester	C_46_H_74_O_14_	TRG	Zhou et al. (2016)
83	Zingibroside R1-6′-butyl ester	C_46_H_74_O_14_	TRG	Zhou et al. (2016)
84	Zingibroside R1-6′-methyl ester	C_43_H_68_O_14_	TRG	Zhou et al. (2016)
85	Oleanolic acid- 28-Oleanolic acid- 28-O- beta- D- Glucopyranoside	C_36_H_58_O_8_	TRG	Xu et al. (2016)
86	20(*S*),25(*R*)-epoxydammarane- 3b,12b,24b,26-tetraol	C_30_H_52_O_5_	TRG	Zheng et al. (2016)
87	20(*S*),25-epoxydammarane-3b,12b,24a-triol	C_30_H_52_O_4_	TRG	Zheng et al. (2016)
88	Stipuleanoside R1	C_47_H_74_O_18_	TRG	Xu et al. (2016)
89	Cynarasaponin C	C_35_H_70_O_19_	TRG	Xu et al. (2016)
90	Spinasaponin A	C_35_H_70_O_19_	TRG	Xu et al. (2016)
91	Quadrangulcoside	C_54_H_90_O_23_	TRG	Xu et al. (2016)
92	Melilotoside	C_41_H_68_O_12_	TRG	Xu et al. (2016)
93	Hebevinoside VI	C_41_H_68_O_12_	TRG	Xu et al. (2016)
94	Quinquenoside F1	C_42_H_74_O_15_	TRG	Xu et al. (2016)
95	Ursan-3b,19a,22b-triol-3-O-b-D-glucopyranosyl (2'→1″)-b-D-glucopyranoside	C_42_H_72_O_13_	TRG	Chung et al. (2014)
96	Ursan-3a,11b-diol-3-O-a-D-glucopyranosyl-(6'→1″)-a-D-glucopyranosyl-(6''→1‴)-a-D-glucopyranosyl-(6‴→1‴')-a-D-glucopyranoside	C_54_H_92_O_22_	TRG	Chung et al. (2014)
97	Lanost-5,24-dien-3b-ol-3-O-b-D-glucopyranosyl-(6'→1″)-b-D-glucopyranosyl-(6''→1‴)-b-D-glucopyranoside	C_48_H_80_O_16_	TRG	Chung et al. (2014)

The structure of ginsenoside is directly related to anticancer activities. Interestingly, in red ginseng processing, the position of sugar chain hydrolysis (carbon-3 > carbon-6 > carbon-20) results in different degrees of anticancer activity (Kai et al., 2015). The 20(*S*)-ginsenosides have more substantial anticancer potential than their 20(*R*)-stereoisomers (Wang et al., 2007; Seoyoung et al., 2009). Ginsenosides with a double bond at C-20 (21) exhibit more effective anticancer activities than those at C-20 (22) (Kai, et al., 2015).

During red ginseng processing, the polar compounds transform into less polar compounds, which exhibit different pharmacological activity from other red ginseng products. TRG, SG, BG, FRG, and PRG have many ginsenosides in common, such as the major ginsenosides Rb1, Rb2, Rc, Rd, Re, and Rg1. In contrast, the minor ginsenosides are different in content and variety. So, red ginseng product pharmacological activity is significantly different from the diverse ingredients.

### 3.2 Transformation of ginsenosides

Ginsenosides can cause a structure change during red ginseng processing. Malonyl-ginsenoside is an initial form of ginseng before processing, after which deglycosylation, decarboxylation, and dehydration will occur. For example, Rg2, Rh1, and Rg3 are deglycosylated from Re, Rg1, and Rd; Rg3 and Rg2 are dehydrated to form Rg5 and Rg6; and malonyl-ginsenoside Rb2 and Rc are decarboxylated to create Rs1 and Rs2 (Chen et al., 2020). Previous research has indicated that ginsenoside Rb1 and Rb2 can transform into ginsenoside Rg3, and ginsenoside Re can transform into ginsenoside Rg2 (Lee et al., 2008; Kim Y. J. et al., 2014). The Maillard reaction is also the primary chemical reaction in red ginseng processing, whereby products are generated whenever reducing sugars are heated with amino acids, peptides, or proteins (Chen and Kitts, 2012). These products are a significant source of compounds that enhance antioxidant activity through heat treatment (Chen and Kitts, 2011). In the Maillard reaction, ginsenosides provide the reducing sugars and then transform into other ginsenosides (Yamabe et al., 2013).

Before being processed into red ginseng, malonyl-20(*S*)-ginsenoside is a natural compound of ginseng. The deglycosylate in carbon-20 easily occurs after processing; however, the location of the 20(*S*) and 20(*R*) epimers remains to be determined. The 20(*R*)-ginsenosides are present through the OH group selective attack after the glycosyl residue elimination at carbon-20 during red ginseng processing (Kang et al., 2007). For example, the ginsenoside Rg3, Rh2, Rs3, Rg2, Rh1, and Rf both have the 20(*S*) and 20(*R*) epimers.

**FIGURE 3:**
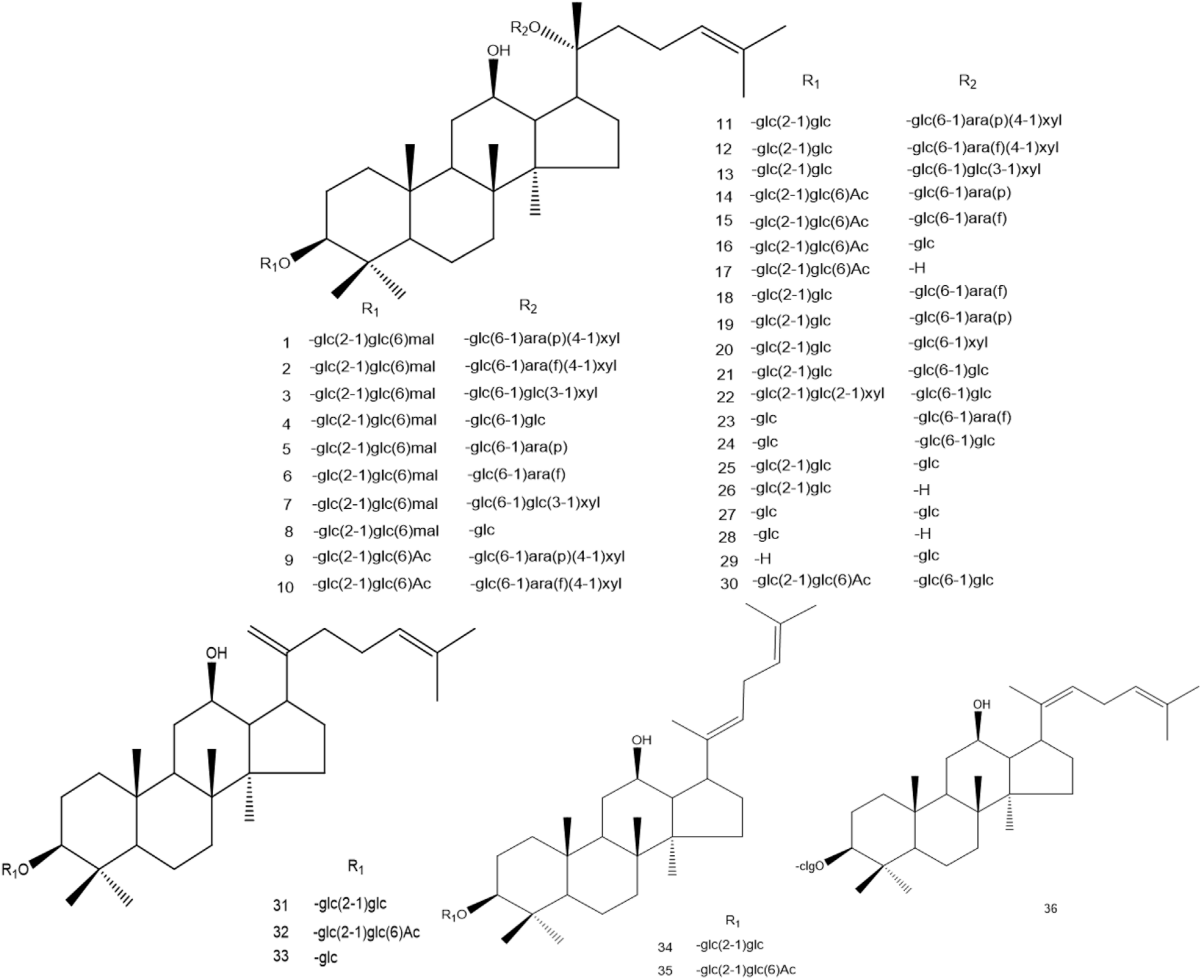
Structure of protopanaxadiol ginsenosides. (ara(f):*α-L*-arabinofuranosyl; ara(p):*α-L*-arabinopyranosyl; rha:*α-L*-ahamnopyranosyl; glc:*β-D*-glucopyranosyl; xyl:*β-D*-xylopyranosyl; mal:malonyl; Ac:acetyl).

**FIGURE 4:**
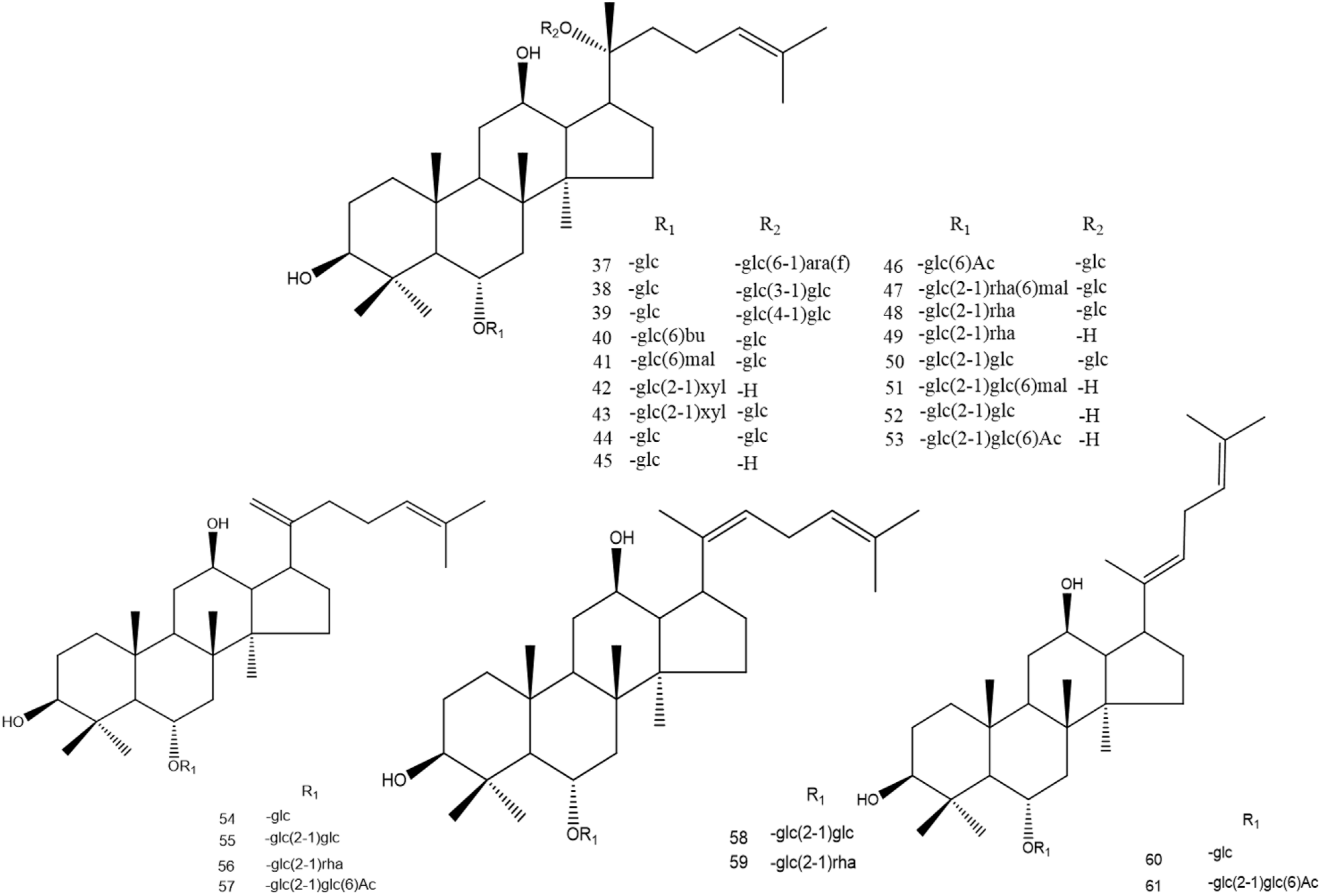
Structure of protopanaxatriol ginsenosides. (ara(f):*α-L*-arabinofuranosyl; rha:*α-L*-ahamnopyranosyl; glc:*β-D*-glucopyranosyl; xyl:*β-D*-xylopyranosyl; mal:malonyl; bu:butyl; Ac:acetyl).

**FIGURE 5:**
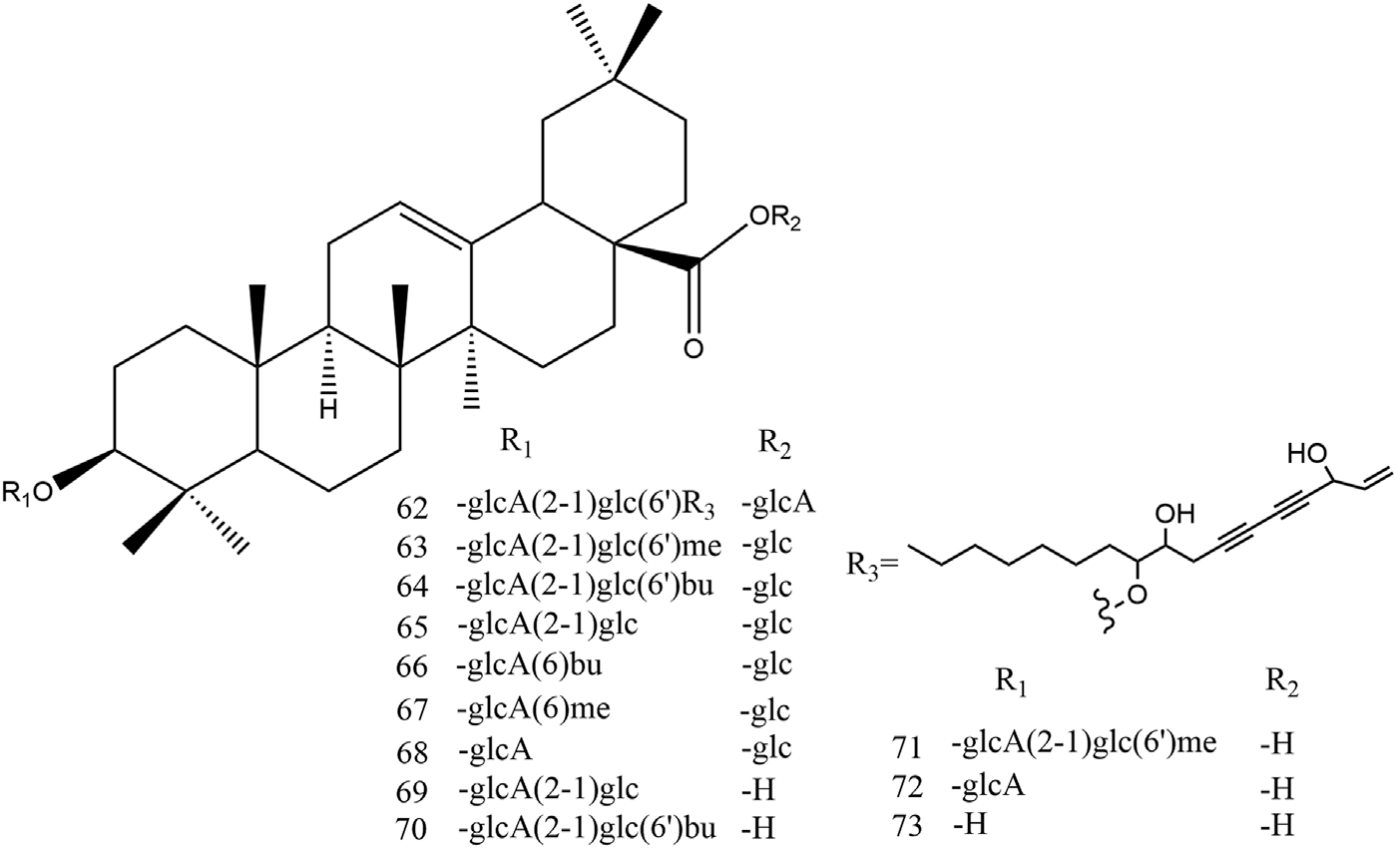
Structure of oleanolic acid ginsenosides. (glcA: *β-D*-glucuronosyl; glc: *β-D*-glucopyranosyl; me: methyl; bu: butyl).

**FIGURE 6:**
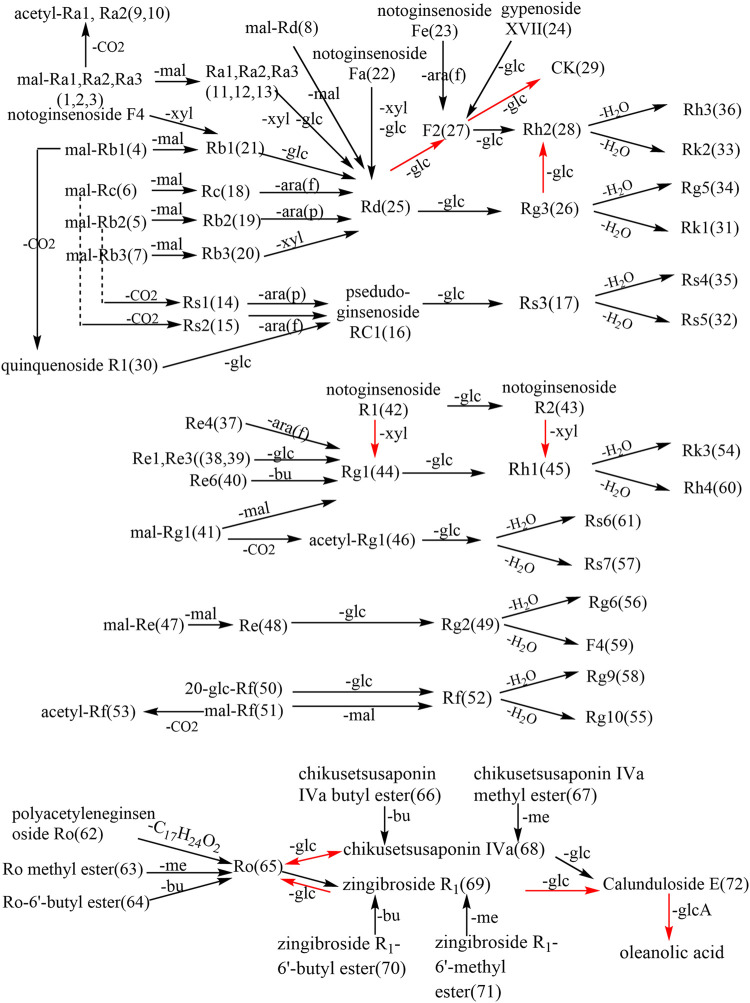
Transformation of ginsenosides in red ginseng products. (ara(f):*α-L*-arabinofuranosyl; ara(p):*α-L*-arabinopyranosyl; rha:*α-L*-ahamnopyranosyl; glc:*β-D*-glucopyranosyl; glcA: *β-D*-glucuronosyl; xyl:*β-D*-xylopyranosyl; mal:malonyl; bu: butyl. The red arrow represents biotransformation).

TRG processing is mainly conducted by decarbonylation, decarboxylation, and the first deglycosylation in carbon-20 of the dammarane skeleton. SG, BG, and PRG promote these reactions. The second deglycosylation is mainly conducted in carbon-20 of the dammarane skeleton and dehydration in this position. However, the FRG processes some characteristic ginsenosides, such as ginsenoside CK, for microbial metabolism. At the same time, the amount of some minor ginsenosides, such as ginsenoside Rh2, Rg1, and Rh1, also increase with the fermentation process. The conversion pathway of oleanane ginsenosides is similar to dammarane ginsenosides. The demethylation, debutylization, and deglycosylation of ester bonds can easily occur at carbon-3 and carbon-28. The ether bond is stable at carbon-3 in the oleanane skeleton, which is also stable between sugars in this position (Figures 3–6).

## 4 20(*R*)- and 20(*S*)-ginsenoside isomerism

Ginsenosides, active ingredients of *P. ginseng*, like 20(*R*)-ginsenoside and 20(*S*)-ginsenoside, exist as stereoisomers depending on the position of the hydroxyl group on carbon-20. A literature survey shows that ginsenoside Rg3 and Rh2 are stereospecific in the stimulation of the pharmacological activity. 20 (*S*)-ginsenoside is the dominant conformation relative to 20(*R*)-ginsenoside in terms of some activity experiments.

In an *in vitro* study, the stereochemistry of the hydroxyl group at C-20 may play an important role in preventing rotavirus infection, and anticancer and osteoclastogenesis inhibitory activity. Ginsenoside Rb2 and its hydrolytic product; 20(*S*)-ginsenoside Rg3, but not 20(*R*)-ginsenoside Rg3, prevent rotavirus infection (Yang et al., 2018). The regulation of DNA methylation may play an important role in the inhibitory effect of ginsenoside Rg3 on the growth of the HepG2 cell line. The inhibitory effect of 20(*S*)-ginsenoside Rg3 is stronger than that of 20(*R*)-ginsenoside Rg3 (Teng et al., 2017). 20(*S*)-Ginsenoside Rh2 potently protects HepG2 cells cytotoxicity treated with tert-butyl hydroperoxide, but 20(*S*)-ginsenoside Rg3 weakly protects it (Lee et al., 2005). The ginsenoside 20(*R*)-ginsenoside Rh2, but not ginsenoside 20(*S*)-ginsenoside Rh2, shows selective osteoclastogenesis inhibitory activity without any cytotoxicity on osteoclastogenesis using RAW264 cells (Liu et al., 2009).

During *in vivo* experiments, ginsenoside isomers showed significant differences in improving immunity, survival ability, and alleviating diabetes symptoms. 20(*R*)- ginsenoside Rg3 has a more potent adjuvant activity of the immune response than 20(*S*)-ginsenoside Rg3 with highly upregulated serum IFN-γ and IL-5 (Wei et al., 2012). The novel characteristics of 20(*S*)-ginsenoside Rg3 exhibited higher pharmacological effects in insulin secretion and AMPK activation than 20(*R*)-ginsenoside Rg3, suggesting that ginsenoside Rg3 epimers show differential activities; 20(*S*)- ginsenoside Rg3 may be a valuable candidate for an anti-diabetic agent (Park et al., 2008). 20(*S*)-ginsenoside Rg3 promotes angiogenesis by activating the AKT/ERK-eNOS signal pathway, and its activity is significantly more potent than that of 20(*R*)-ginsenoside Rg3 (Kwok et al., 2012). In addition, ginsenosides can also maintain body growth with a supplement of 20(*S*)-, but not 20(*R*)-ginsenoside Rg3 in a cholesterol-deprived medium (Lee et al., 2011).

The hydroxyl stereochemistry at C-20 has a great influence on the activity of ginsenosides. In the production process, the processing method of red ginseng should be changed according to the actual demand to achieve the maximum transformation of the target ginsenosides. For example, under optimum reaction conditions, the actual 20(*R*)-ginsenoside Rg3 converts PPD ginsenosides (Sun et al., 2013). As mentioned, ginsenoside Rg3, Rh2, Rs3, Rg2, Rh1, and Rf have the 20(*S*) and 20(*R*) epimers. There are few studies regarding the structure-activity relationship of other ginsenosides, which is still an urgent problem to be solved. In the future, these problems should be explored to determine the greater value of red ginseng.

## 5 Clinical trials of red ginseng

**FIGURE 7:**
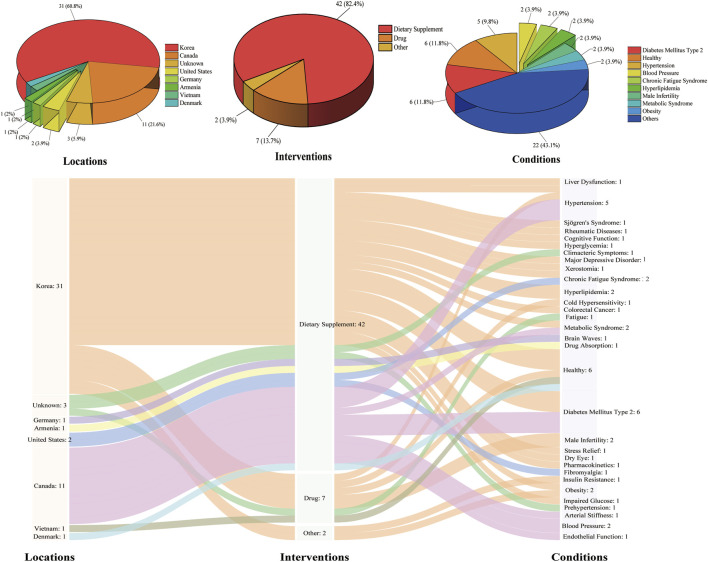
Analysis of clinical trials of red ginseng.

ClinicalTrials.gov is a database of privately and publicly funded clinical studies conducted worldwide. Through the ClinicalTrials.gov platform (https://clinicaltrials.gov/), a search of the term “red ginseng” found 78 research projects; while the exclusion of all other forms of ginseng other than red ginseng obtained 39 clinical studies (Figure 7); only six of these research projects were successfully concluded and the results were reported. These results suggest that KRG is efficacious as an adjuvant treatment for patients experiencing residual symptoms of major depression (Jeong et al., 2015), reducing proinflammatory cytokines and fatigue in overweight patients with non-alcoholic fatty liver disease (Hong et al., 2016), and protecting subjects from contracting acute respiratory illness (Lee S. A. et al., 2012). KRG may have beneficial effects for dry mouth in women, especially in those of menopausal age, but not in men (Park et al., 2010). There is no evidence that KRG has an effect on blood pressure, fasting blood glucose, or arterial stiffness in subjects with metabolic syndrome (Park et al., 2012). Based on mostly low certainty evidence, ginseng may only have trivial effects on erectile function or satisfaction with intercourse compared to a placebo when assessed using validated instruments (Lee Y. S. et al., 2021). South Korea and Canada lead the studies. Twenty-eight institutions from seven countries participated, including the Clinical Trial Center for Functional Foods Chonbuk National University Hospital, Clinical Nutrition and Risk Factor Modification Centre, and Clinical Trial Center for Functional Foods. Red ginseng is mainly used as a dietary supplement, and a small part is used in drug research. There are 31 research directions, primarily focusing on diabetes mellitus type 2, health, hypertension, and blood pressure, etc. Given this, clinical studies of red ginseng mainly focus on Korea and Canada and the use of dietary supplements in human clinical studies of type II diabetes, health, hypertension, and other neurological diseases.

## 6 Discussion

Red ginseng products derived from fresh ginseng include TRG, SG, BG, FRG, and PRG. Their chemical compositions are similar because all of them were derived from the same original plant, *P. ginseng*. The chemical composition of these products has been extensively reported. However, the research mainly focuses on TRG. The chemical composition differences between other red ginseng products remain undiscovered. Interestingly, the transformation law of ginsenosides in different red ginseng processing is similar. Some identical rare ginsenosides make other red ginseng products with similar pharmacological activities. For example, ginsenosides (Rg3, Rg5, and Rk1) of SG have been reported to improve cognitive function; ginsenosides (Rg3, Rg5, and Rk1) of BG are the same as SG, and many studies have been conducted on the prevention and treatment of obesity, breast cancer, cognitive impairment, and fetal a syndrome, etc. The composition of FRG by microorganisms is similar to that of other red ginseng products, which convert normal ginsenosides into minor ginsenosides and produce a unique component CK, which is mainly used in improving allergies, diabetes, anxiety, and other aspects, and may play an essential role in metabolic syndrome and other elements. PRG significantly reduces the conversion time of normal ginsenosides into minor ginsenosides. According to current *in vitro* studies, the anticancer effects of PRG are worthy of further studies.

Unfortunately, chemical constituents and efficacy studies on red ginseng products are still lacking, especially for PRG. The pharmacological activities focus on TRG and another new type of red ginseng. In some pharmacological activities, the new kind of red ginseng is more vital than TRG. There is little systematic research comparing the differences in pharmacological activities between the new types of red ginseng, and further studies should be conducted on the mechanism of pharmacodynamics in different red ginseng products applied appropriately in the clinic.

During red ginseng processing, the polar compounds are transformed into less polar compounds; demalonylation and decarboxylation easily occur, and the malonyl-ginsenosides decarboxylate into acetyl-ginsenosides; the Maillard reaction is also a reaction in red ginseng processing (Figure 1). The different process influences the content and variety of minor ginsenosides. Usually, the ginsenoside transformation of the new type of red ginseng is more vital than TRG. So, the amount of minor ginsenosides is minimal in TRG but abundant in the new type of red ginseng. With a further understanding of the structure and efficacy of ginsenosides, rare ginsenosides, such as Rg3 and Rh2, have been found to have significant pharmacological activities. The species and proportion of ginsenosides are different during red ginseng processing, which suggests that processing is crucial for the efficacy of red ginseng products. The processing of TCM expands the scope of the application thereof, which is the charm of TCM.

## 7 Conclusion

Modern technology applied to red ginseng processing provides further specifications. At the same time, modern technology can adapt to determine the development law of modern diseases. Red ginseng products are used in the treatment of AD, diabetes, and other conditions, and further systematic comparative studies of the chemical composition can promote full utilization thereof. The types and proportions of rare ginsenosides require further in-depth comparative analyses in pharmacological activity for better selection of drugs according to different clinical needs. Many researchers are focused on red ginseng processing, especially with regards the steam method and the detailed parameters of processing. In contrast, FRG has the advantage of microbial transformation in the ginsenosides to enhance the amount and variety of minor ginsenosides. To accurately control the processing conditions of different red ginseng products, we can maintain the transformation of ginsenosides and produce red ginseng products for specific diseases. This difference in ginsenosides results in red ginseng products being selectively applied in the clinic.

The processing of red ginseng can be developed in the direction of accurate processing and precise treatment. Moreover, ginsenoside transformation of red ginseng and the structure–functional relationship of ginsenoside derivatives can illustrate the medicinal compositions of this different pharmacological activity, which make the application of red ginseng practical and facilitate the development of new types of red ginseng in the future.

## Abbreviations

TRG, Traditional red ginseng; SG, Sun ginseng; BG, Black ginseng; FRG, Fermented red ginseng; PRG, Puffed red ginseng; WG, White ginseng; *P. ginseng* A.C. Meyer, *Panax ginseng*; KRG, Korean red ginseng; TCM, Traditional Chinese medicine; CK, Compound K; PPD, Protopanaxadiol; PPT, Protopanaxatriol; OLE, Oleanane.

## References

[B1] An Y. E. Ahn S. C. Yang D. C. Park S. J. Kim B. Y. Baik M. Y. (2011). Chemical conversion of ginsenosides in puffed red ginseng. Lwt-Food Sci. Technol. 44 (2), 370–374. 10.1016/j.lwt.2010.09.013

[B2] Bae C. H. Kim J. Nam W. Kim H. Kim J. Nam B. (2021). Fermented red ginseng alleviates ovalbumin-induced inflammation in mice by suppressing interleukin-4 and immunoglobulin E expression. J. Med. Food 24 (6), 569–576. 10.1089/jmf.2020.4854 34161163

[B3] Bae E. A. Han M. J. Kim E. J. Kim D. H. (2004). Transformation of ginseng saponins to ginsenoside Rh-2 by acids and human intestinal bacteria and biological activities of their transformants. Arch. Pharm. Res. 27(1), 61–67. 10.1007/Bf02980048 14969341

[B4] Bae S. H. Lee H. S. Kim M. R. Kim S. Y. Kim J. M. Suh H. J. (2011). Changes of ginsenoside content by mushroom mycelial fermentation in red ginseng extract. J. Ginseng Res. 35 (2), 235–242. 10.5142/jgr.2011.35.2.235 23717066 PMC3659518

[B5] Baek N. I. Kim D. S. Lee Y. H. Park J. D. Lee C. B. Kim S. I. (1996). Ginsenoside Rh4, a genuine dammarane glycoside from Korean red ginseng. Planta Med. 62 (1), 86–87. 10.1055/s-2006-957816 8720394

[B6] Baek N. I. Kim J. M. Park J. H. Ryu J. H. Kim D. S. Lee Y. H. (1997). Ginsenoside Rs(3), a genuine dammarane-glycoside from Korean red ginseng. Arch. Pharm. Res. 20 (3), 280–282. 10.1007/BF02976158 18975165

[B7] Chen W. Balan P. Popovich D. G. (2020). Changes of ginsenoside composition in the creation of black ginseng leaf. Molecules 25 (12), 2809. 10.3390/molecules25122809 32570758 PMC7355439

[B8] Chen X. M. Kitts D. D. (2011). Antioxidant and anti-inflammatory activities of Maillard reaction products isolated from sugar-amino acid model systems. J. Agric. Food Chem. 59 (20), 11294–11303. 10.1021/jf2031583 21936573

[B9] Chen X. M. Kitts D. D. (2012). Characterization of antioxidant and anti-inflammatory activities of bioactive fractions recovered from a glucose-lysine Maillard reaction model system. Mol. Cell Biochem. 364 (1-2), 147–157. 10.1007/s11010-011-1213-7 22234502

[B10] Cho J. G. Lee D. Y. Shrestha S. Lee S. K. Kang H. M. Son S. H. (2013) Three new ginsenosides from the heat-processed roots of Panax ginseng Chem. Nat. Compd. 49 (5), 882–887. 10.1007/s10600-013-0769-8

[B11] Choi I. D. Ryu J. H. Lee D. E. Lee M. H. Shim J. J. Ahn Y. T. (2016) Enhanced absorption study of ginsenoside compound K (20-O-beta-(D-Glucopyranosyl)-20(S)-protopanaxadiol) after oral administration of fermented red ginseng extract (HYFRG) in healthy Korean volunteers and rats. Evid. Based Complement. Altern. Med. 2016, 3908142. 10.1155/2016/3908142 PMC4969531 27516803

[B12] Choi Y. J. Kang L. J. Lee S. G. (2014). Stimulation of DDX3 expression by ginsenoside Rg3 through the Akt/p53 pathway activates the innate immune response via TBK1/IKKε/IRF3 signalling. Curr. Med. Chem. 21 (8), 1050–1060. 10.2174/09298673113206660306 24180280

[B13] Chung I. M. Kim Y. O. Ali M. Kim S. H. Park I. Kim E. H. (2014). Triterpene glycosides from red ginseng marc and their anti-inflammatory activities. Bioorg Med. Chem. Lett. 24 (17), 4203–4208. 10.1016/j.bmcl.2014.07.042 25106885

[B14] Han S. K. Joo M. K. Kim J. K. Jeung W. Kang H. Kim D. H. (2020). Bifidobacteria-fermented red ginseng and its constituents ginsenoside Rd and protopanaxatriol alleviate anxiety/depression in mice by the amelioration of gut dysbiosis. Nutrients 12 (4), 901. 10.3390/nu12040901 32224881 PMC7230967

[B15] Hasegawa H. (2004). Proof of the mysterious efficacy of ginseng: Basic and clinical trials: Metabolic activation of ginsenoside: Deglycosylation by intestinal bacteria and esterification with fatty acid. J. Pharmacol. Sci. 95 (2), 153–157. 10.1254/jphs.fmj04001x4 15215638

[B16] Heo J. H. Lee S. T. Chu K. Oh M. J. Park H. J. Shim J. Y. (2012). Heat-processed ginseng enhances the cognitive function in patients with moderately severe Alzheimer's disease. Nutr. Neurosci. 15 (6), 278–282. 10.1179/1476830512Y.0000000027 22780999

[B17] Hong M. Lee Y. H. Kim S. Suk K. T. Bang C. S. Yoon J. H. (2016). Anti-inflammatory and antifatigue effect of Korean Red Ginseng in patients with nonalcoholic fatty liver disease. J. Ginseng Res. 40 (3), 203–210. 10.1016/j.jgr.2015.07.006 27616896 PMC5005313

[B18] Hoseney R. C. (1986). Principles of cereal science and technology. A general reference cereal foods 51, 415.

[B19] Irfan M. Lee Y. Y. Lee K. J. Kim S. D. Rhee M. H. (2022). Comparative antiplatelet and antithrombotic effects of red ginseng and fermented red ginseng extracts. J. Ginseng Res. 46 (3), 387–395. 10.1016/j.jgr.2021.05.010 35600768 PMC9120646

[B20] Jang D. J. Lee M. S. Shin B. C. Lee Y. C. Ernst E. (2008). Red ginseng for treating erectile dysfunction: A systematic review. Br. J. Clin. Pharmacol. 66 (4), 444–450. 10.1111/j.1365-2125.2008.03236.x 18754850 PMC2561113

[B21] Jang S. H. Park J. Kim S. H. Choi K. M. Ko E. S. Cha J. D. (2017). Red ginseng powder fermented with probiotics exerts antidiabetic effects in the streptozotocin-induced mouse diabetes model. Pharm. Biol. 55 (1), 317–323. 10.1080/13880209.2016.1237978 27927080 PMC6130625

[B22] Jeong H. G. Ko Y. H. Oh S. Y. Han C. Kim T. Joe S. H. (2015). Effect of Korean Red Ginseng as an adjuvant treatment for women with residual symptoms of major depression. Asia Pac Psychiatry 7 (3), 330–336. 10.1111/appy.12169 25504813

[B23] Jiang G. Ramachandraiah K. Murtaza M. A. Wang L. Li S. Ameer K. (2021). Synergistic effects of black ginseng and aged garlic extracts for the amelioration of nonalcoholic fatty liver disease (NAFLD) in mice. Food Sci. Nutr. 9 (6), 3091–3099. 10.1002/fsn3.2267 34136174 PMC8194913

[B24] Jiao L. Zhang X. Wang M. Li B. Liu Z. Liu S. (2014). Chemical and antihyperglycemic activity changes of ginseng pectin induced by heat processing. Carbohydr. Polym. 114, 567–573. 10.1016/j.carbpol.2014.08.018 25263928

[B25] Jung J. Jang H. J. Eom S. J. Choi N. S. Lee N. K. Paik H. D. (2019). Fermentation of red ginseng extract by the probiotic Lactobacillus plantarum KCCM 11613P: Ginsenoside conversion and antioxidant effects. J. Ginseng Res. 43 (1), 20–26. 10.1016/j.jgr.2017.07.004 30662290 PMC6323145

[B26] Kai Q. Liu Q. Wan J. Y. Zhao Y. J. Guo R. Z. Alolga R. N. (2015). Rapid preparation of rare ginsenosides by acid transformation and their structure-activity relationships against cancer cells. Sci. Rep. 5, 8598. 10.1038/srep08598 25716943 PMC4341195

[B27] Kang K. S. Kim H. Y. Pyo J. S. Yokozawa T. (2006). Increase in the free radical scavenging activity of ginseng by heat-processing. Biol. Pharm. Bull. 29 (4), 750–754. 10.1248/bpb.29.750 16595912

[B28] Kang K. S. Tanaka T. Cho E. J. Yokozawa T. (2009) Evaluation of the peroxynitrite scavenging activity of heat-processed ginseng J. Med. Food 12 (1), 124–130. 10.1089/jmf.2007.0646 19298205

[B29] Kang K. S. Yamabe N. Kim H. Y. Okamoto T. Sei Y. Yokozawa T. (2007). Increase in the free radical scavenging activities of American ginseng by heat processing and its safety evaluation. J. Ethnopharmacol. 113 (2), 225–232. 10.1016/j.jep.2007.05.027 17618072

[B30] Kasai R. Besso H. Tanaka O. Saruwatari Y. Fuwa T. (1983). Saponins of red ginseng. Chem. Pharm. Bull. (Tokyo) 31 (6), 2120–2125. 10.1248/cpb.31.2120

[B31] Kennedy D. O. Scholey A. B. (2003). Ginseng: Potential for the enhancement of cognitive performance and mood. Pharmacol. Biochem. Behav. 75 (3), 687–700. 10.1016/s0091-3057(03)00126-6 12895687

[B32] Kho M. C. Lee Y. J. Park J. H. Kim H. Y. Yoon J. J. Ahn Y. M. (2016). Fermented red ginseng potentiates improvement of metabolic dysfunction in metabolic syndrome rat models. Nutrients 8 (6), 369. 10.3390/nu8060369 27322312 PMC4924210

[B33] Kim A. J. (2015). Physiological activities of 9 cycle steaming and drying black ginseng using Makgeolli. Food Sci. Biotechnol. 24 (5), 1867–1873. 10.1007/s10068-015-0244-3

[B34] Kim D. S. Baek N. I. Lee Y. H. Park J. D. Kim S. I. (1996). Preparation and structure determination of a new glycoside, (20E)-ginsenoside Rh3, and its isomer from dioltype ginseng saponins. Yakhak Hoeji 39, 86–93.

[B35] Kim H. I. Kim J. K. Kim J. Y. Han M. J. Kim D. H. (2019). Fermented red ginseng and ginsenoside Rd alleviate ovalbumin-induced allergic rhinitis in mice by suppressing IgE, interleukin-4, and interleukin-5 expression. J. Ginseng Res. 43 (4), 635–644. 10.1016/j.jgr.2019.02.006 31695569 PMC6823749

[B36] Kim I. K. Lee K. Y. Kang J. Park J. S. Jeong J. (2021). Immune-modulating effect of Korean red ginseng by balancing the ratio of peripheral T lymphocytes in bile duct or pancreatic cancer patients with adjuvant chemotherapy. Vivo 35 (3), 1895–1900. 10.21873/invivo.12454 PMC8193343 33910879

[B37] Kim J. H. Ahn S. C. Choi S. W. Hur N. Y. Kim B. Y. Baik M. Y. (2008). Changes in effective components of ginseng by puffing. J. Korean Soc. Appl. Biol. Chem. 51 (3), 188–193.

[B38] Kim J. H. Han I. H. Yamabe N. Kim Y. J. Lee W. Eom D. W. (2014). Renoprotective effects of Maillard reaction products generated during heat treatment of ginsenoside Re with leucine. Food Chem. 143, 114–121. 10.1016/j.foodchem.2013.07.075 24054220

[B39] Kim J. K. Choi M. S. Jeung W. Ra J. Yoo H. H. Kim D. H. (2020). Effects of gut microbiota on the pharmacokinetics of protopanaxadiol ginsenosides Rd, Rg3, F2, and compound K in healthy volunteers treated orally with red ginseng. J. Ginseng Res. 44 (4), 611–618. 10.1016/j.jgr.2019.05.012 32617041 PMC7322745

[B40] Kim J. K. Kim J. Y. Jang S. E. Choi M. S. Jang H. M. Yoo H. H. (2018). Fermented red ginseng alleviates cyclophosphamide-induced immunosuppression and 2,4,6-trinitrobenzenesulfonic acid-induced colitis in mice by regulating macrophage activation and T cell differentiation. Am. J. Chin. Med. 46 (8), 1879–1897. 10.1142/S0192415X18500945 30518233

[B41] Kim M. O. Lee J. W. Lee J. K. Song Y. N. Oh E. S. Ro H. (2022). Black ginseng extract suppresses airway inflammation induced by cigarette smoke and lipopolysaccharides *in vivo* . Antioxidants (Basel) 11 (4), 679. 10.3390/antiox11040679 35453364 PMC9025275

[B42] Kim S. J. Kim A. K. (2015). Anti-breast cancer activity of fine black ginseng (Panax ginseng meyer) and ginsenoside Rg_5_ . J. Ginseng Res. 39 (2), 125–134. 10.1016/j.jgr.2014.09.003 26045685 PMC4452536

[B43] Kim W. Y. Kim J. M. Han S. B. Lee S. K. Kim N. D. Park M. K. (2000). Steaming of ginseng at high temperature enhances biological activity. J. Nat. Prod. 63 (12), 1702–1704. 10.1021/np990152b 11141123

[B44] Kim Y. J. Choi W. I. Jeon B. N. Choi K. C. Kim K. Kim T. J. (2014). Stereospecific effects of ginsenoside 20-Rg3 inhibits TGF-β1-induced epithelial-mesenchymal transition and suppresses lung cancer migration, invasion and anoikis resistance. Toxicology 322, 23–33. 10.1016/j.tox.2014.04.002 24793912

[B45] Kitagawa I. Yoshikawa M. Yoshihara M. Hayashi T. Taniyama T. (1983). Chemical studies on crude drug precession. I. On the constituents of ginseng radix rubra (1). Yakugaku Zasshi-journal Pharm. Soc. Jpn. 103 (6), 612–622. 10.1248/yakushi1947.103.6_612 6655550

[B46] Kwok H. H. Guo G. L. Lau J. K. Cheng Y. K. Wang J. R. Jiang Z. H. (2012). Stereoisomers ginsenosides-20(S)-Rg_3_ and -20(R)-Rg_3_ differentially induce angiogenesis through peroxisome proliferator-activated receptor-gamma. Biochem. Pharmacol. 83 (7), 893–902. 10.1016/j.bcp.2011.12.039 22234331

[B47] Kwon S. W. Han S. B. Park I. H. Kim J. M. Park M. K. Park J. H. (2001). Liquid chromatographic determination of less polar ginsenosides in processed ginseng. J. Chromatogr. A 921 (2), 335–339. 10.1016/s0021-9673(01)00869-x 11471818

[B48] Lee B. Sur B. Lee H. Oh S. (2020). Korean Red Ginseng prevents posttraumatic stress disorder-triggered depression-like behaviors in rats via activation of the serotonergic system. J. Ginseng Res. 44 (4), 644–654. 10.1016/j.jgr.2019.09.005 32617045 PMC7322749

[B49] Lee C. H. Kim J. M. Kim D. H. Park S. J. Liu X. Cai M. (2013). Effects of Sun ginseng on memory enhancement and hippocampal neurogenesis. Phytother. Res. 27 (9), 1293–1299. 10.1002/ptr.4873 23109250

[B50] Lee C. S. Lee J. H. Oh M. Choi K. M. Jeong M. R. Park J. D. (2012). Preventive effect of Korean red ginseng for acute respiratory illness: A randomized and double-blind clinical trial. J. Korean Med. Sci. 27 (12), 1472–1478. 10.3346/jkms.2012.27.12.1472 23255845 PMC3524425

[B51] Lee H. Lee J. Y. Song K. C. Kim J. Park J. H. Chun K. H. (2012). Protective effect of processed Panax ginseng, sun ginseng on UVB-irradiated human skin keratinocyte and human dermal fibroblast. J. Ginseng Res. 36 (1), 68–77. 10.5142/jgr.2012.36.1.68 23717106 PMC3659574

[B52] Lee H. U. Bae E. A. Han M. J. Kim D. H. (2005). Hepatoprotective effect of 20(S)-ginsenosides Rg3 and its metabolite 20(S)-ginsenoside Rh2 on tert-butyl hydroperoxide-induced liver injury. Biol. Pharm. Bull. 28 (10), 1992–1994. 10.1248/bpb.28.1992 16204963

[B53] Lee H. W. Lee M. S. Kim T. H. Alraek T. Zaslawski C. Kim J. W. (2021). Ginseng for erectile dysfunction. Cochrane Database Syst. Rev. 4 (4), CD012654. 10.1002/14651858.CD012654.pub2 33871063 PMC8094213

[B54] Lee J. H. Ahn J. Y. Shin T. J. Choi S. H. Lee B. H. Hwang S. H. (2011). Effects of minor ginsenosides, ginsenoside metabolites, and ginsenoside epimers on the growth of *Caenorhabditis elegans* . J. Ginseng Res. 35 (3), 375–383. 10.5142/jgr.2011.35.3.375 23717083 PMC3659541

[B55] Lee S. Jung S. You H. Lee Y. Park Y. Lee H. (2022). Effect of fermented red ginseng concentrate intake on stool characteristic, biochemical parameters, and gut microbiota in elderly Korean women. Nutrients 14 (9), 1693. 10.3390/nu14091693 35565660 PMC9105854

[B56] Lee S. A. Jo H. K. Im B. O. Kim S. Whang W. K. Ko S. K. (2012). Changes in the contents of prosapogenin in the red ginseng (Panax ginseng) depending on steaming batches. J. Ginseng Res. 36 (1), 102–106. 10.5142/jgr.2012.36.1.102 23717110 PMC3659570

[B57] Lee S. H. Oh M. Park J. Jang S. Y. Cheong S. H. Lee H. (2015). Effects of µ-opioid receptor gene polymorphism on postoperative nausea and vomiting in patients undergoing general anesthesia with remifentanil: Double blinded randomized trial. Food Sci. Biotechnol. 24 (2), 651–657. 10.3346/jkms.2015.30.5.651 PMC4414652 25931799

[B58] Lee S. J. Lee D. Y. O'Connell J. F. Egan J. M. Kim Y. (2022). Black ginseng ameliorates cellular senescence via p53-p21/p16 pathway in aged mice. Biol. (Basel) 11 (8), 1108. 10.3390/biology11081108 PMC9331701 35892965

[B59] Lee S. J. Oh S. Kim M. J. Sim G. S. Moon T. W. Lee J. (2018). Oxidative stability of extracts from red ginseng and puffed red ginseng in bulk oil or oil-in-water emulsion matrix. J. Ginseng Res. 42 (3), 320–326. 10.1016/j.jgr.2017.04.002 29983613 PMC6026360

[B60] Lee S. M. Kim S. C. Oh J. Kim J. H. Na M. Seong S. C. (2013a). 20(R)-Ginsenoside Rf: A new ginsenoside from red ginseng extract. Phytochem. Lett. 6 (4), 620–628. 10.1007/s00167-012-1998-2

[B61] Lee S. M. Seo H. K. Oh J. Na M. (2013b). Updating chemical profiling of red ginseng via the elucidation of two geometric isomers of ginsenosides Rg9 and Rg10. Food Chem. 141 (4), 3920–3924. 10.1016/j.foodchem.2013.07.012 23993566

[B62] Lee S. R. Kim M. R. Yon J. M. Baek I. J. Park C. G. Lee B. J. (2009). Black ginseng inhibits ethanol-induced teratogenesis in cultured mouse embryos through its effects on antioxidant activity. Toxicol Vitro 23 (1), 47–52. 10.1016/j.tiv.2008.10.001 18992320

[B63] Lee W. Ku S. K. Kim J. E. Cho S. H. Song G. Y. Bae J. S. (2019). Inhibitory effects of black ginseng on particulate matter-induced pulmonary injury. Am. J. Chin. Med. 47 (6), 1237–1251. 10.1142/S0192415X19500630 31495180

[B64] Lee Y. J. Kim H. Y. Kang K. S. Lee J. G. Yokozawa T. Park J. H. (2008). The chemical and hydroxyl radical scavenging activity changes of ginsenoside-Rb1 by heat processing. Bioorg Med. Chem. Lett. 18 (16), 4515–4520. 10.1016/j.bmcl.2008.07.056 18676142

[B65] Lee Y. S. Kim K. W. Yoon D. Kim G. S. Kwon D. Y. Kang O. H. (2021). Comparison of antivirulence activities of black ginseng against methicillin-resistant *Staphylococcus aureus* according to the number of repeated steaming and drying cycles. Antibiot. (Basel) 10 (6), 617. 10.3390/antibiotics10060617 PMC8224340 34064076

[B66] Li B. H. Zhao J. O. Wang C. Z. Searle J. He T. C. Yuan C. S. (2011). Ginsenoside Rh2 induces apoptosis and paraptosis-like cell death in colorectal cancer cells through activation of p53. Cancer Lett. 301 (2), 185–192. 10.1016/j.canlet.2010.11.015 21194832 PMC3022099

[B67] Li J. Zhong W. Wang W. Hu S. Yuan J. Zhang B. (2014). Ginsenoside metabolite compound K promotes recovery of dextran sulfate sodium-induced colitis and inhibits inflammatory responses by suppressing NF-κB activation. PLoS One 9 (2), e87810. 10.1371/journal.pone.0087810 24504372 PMC3913696

[B68] Liu H. Lu X. Hu Y. Fan X. (2020). Chemical constituents of Panax ginseng and Panax notoginseng explain why they differ in therapeutic efficacy. Pharmacol. Res. 161, 105263. 10.1016/j.phrs.2020.105263 33127555

[B69] Liu J. Shiono J. Shimizu K. Yu H. Zhang C. Jin F. (2009). 20(R)-ginsenoside Rh2, not 20(S), is a selective osteoclastgenesis inhibitor without any cytotoxicity. Bioorg Med. Chem. Lett. 19 (12), 3320–3323. 10.1016/j.bmcl.2009.04.054 19428246

[B70] Min J. H. Cho H. J. Yi Y. S. (2022). A novel mechanism of Korean Red Ginseng-mediated anti-inflammatory action via targeting caspase-11 non-canonical inflammasome in macrophages. J. Ginseng Res. 46 (5), 675–682. 10.1016/j.jgr.2021.12.009 36090677 PMC9459075

[B71] Oh H. B. Lee J. W. Lee D. E. Na S. C. Jeong D. E. Hwang D. I. (2021). Characteristics of black ginseng (Panax ginseng C.A. Mayer) production using ginseng stored at low temperature after harvest. Metabolites 11 (2), 98. 10.3390/metabo11020098 33578877 PMC7916568

[B72] Oh J. Jeon S. B. Lee Y. Lee H. Kim J. Kwon B. R. (2015). Fermented red ginseng extract inhibits cancer cell proliferation and viability. J. Med. Food 18 (4), 421–428. 10.1089/jmf.2014.3248 25658580

[B73] Park B. J. Lee Y. J. Lee H. R. Jung D. H. Na H. Y. Kim H. B. (2012). Effects of Korean red ginseng on cardiovascular risks in subjects with metabolic syndrome: A double-blind randomized controlled study. Korean J. Fam. Med. 33 (4), 190–196. 10.4082/kjfm.2012.33.4.190 22916320 PMC3418337

[B74] Park H. J. Shim H. S. Kim K. S. Shim I. (2011). The protective effect of black ginseng against transient focal ischemia-induced neuronal damage in rats. Korean J. Physiol. Pharmacol. 15 (6), 333–338. 10.4196/kjpp.2011.15.6.333 22359470 PMC3282220

[B75] Park I. H. Han S. B. Kim J. M. Piao L. Kwon S. W. Kim N. Y. (2002a). Four new acetylated ginsenosides from processed ginseng (sun ginseng). Arch. Pharm. Res. 25 (6), 837–841. 10.1007/BF02977001 12510835

[B76] Park I. H. Kim N. Y. Han S. B. Kim J. M. Kwon S. W. Kim H. J. (2002b). Three new dammarane glycosides from heat processed ginseng. Arch. Pharm. Res. 25 (4), 428–432. 10.1007/BF02976595 12214849

[B77] Park J. D. Lee Y. H. Kim S. I. (1998). Ginsenoside Rf2, a new dammarane glycoside from Korean red ginseng (Panax ginseng). Arch. Pharm. Res. 21 (5), 615–617. 10.1007/BF02975384 9875504

[B78] Park J. W. Lee B. J. Bu Y. Yeo I. Kim J. Ryu B. H. (2010). Effects of Korean red ginseng on dry mouth: A randomized, double-blind, placebo-controlled trial. J. Ginseng Res. 34 (3), 183–191. 10.5142/jgr.2010.34.3.183

[B79] Park M. W. Ha J. Chung S. H. (2008). 20(S)-ginsenoside Rg3 enhances glucose-stimulated insulin secretion and activates AMPK. Biol. Pharm. Bull. 31 (4), 748–751. 10.1248/bpb.31.748 18379076

[B80] Park S. J. Park M. Sharma A. Kim K. Lee H. J. (2019). Black ginseng and ginsenoside Rb1 promote browning by inducing UCP1 expression in 3T3-L1 and primary white adipocytes. Nutrients 11 (11), 2747. 10.3390/nu11112747 31726767 PMC6893667

[B81] Peng X. Hao M. Zhao Y. Cai Y. Chen X. Chen H. (2021). Red ginseng has stronger anti-aging effects compared to ginseng possibly due to its regulation of oxidative stress and the gut microbiota. Phytomedicine 93, 153772. 10.1016/j.phymed.2021.153772 34753028

[B82] Ryu J. H. Park J. H. Kim T. H. Dong H. S. Kim J. M. Park J. H. (1996). A genuine dammarane glycoside, (20E)-ginsenoside F 4 from Korean red ginseng. Arch. Pharm. Res. 19 (4), 335–336. 10.1007/bf02976251

[B83] Saba E. Lee Y. Y. Kim M. Kim S. H. Hong S. B. Rhee M. H. (2018). A comparative study on immune-stimulatory and antioxidant activities of various types of ginseng extracts in murine and rodent models. J. Ginseng Res. 42 (4), 577–584. 10.1016/j.jgr.2018.07.004 30344431 PMC6191938

[B84] Seoyoung L. Geuntae K. Sihun R. Song J. S. Hiejoon K. Hong S. S. (2009). Proteomic analysis of the anti-cancer effect of 20S-ginsenoside Rg3 in human colon cancer cell lines. Biosci. Biotech. BIOCH 73 (4), 811–816. 10.1271/bbb.80637 19352032

[B85] Shin J. H. Park Y. J. Kim W. Kim D. O. Kim B. Y. Lee H. (2019). Change of ginsenoside profiles in processed ginseng by drying, steaming, and puffing. J. Microbiol. Biotechnol. 29 (2), 222–229. 10.4014/jmb.1809.09056 30609886

[B86] Shin S. J. Park Y. H. Jeon S. G. Kim S. Nam Y. Oh S. M. (2020). Red ginseng inhibits tau aggregation and promotes tau dissociation *in vitro* . Oxid. Med. Cell Longev. 2020, 7829842. 10.1155/2020/7829842 32685100 PMC7350179

[B87] Song K. C. Chang T. S. Lee H. Kim J. Park J. H. Hwang G. S. (2012). Processed Panax ginseng, sun ginseng increases type I collagen by regulating MMP-1 and TIMP-1 expression in human dermal fibroblasts. J. Ginseng Res. 36 (1), 61–67. 10.5142/jgr.2012.36.1.61 23717105 PMC3659568

[B88] Sun B. S. Gu L. J. Fang Z. M. Wang C. Y. Wang Z. Lee M. R. (2009). Simultaneous quantification of 19 ginsenosides in black ginseng developed from Panax ginseng by HPLC-ELSD. J. Pharm. Biomed. Anal. 50 (1), 15–22. 10.1016/j.jpba.2009.03.025 19394786

[B89] Sun C. Gao W. Zhao B. Cheng L. (2013). Optimization of the selective preparation of 20(R)-ginsenoside Rg3 catalyzed by d, l-tartaric acid using response surface methodology. Fitoterapia 84, 213–221. 10.1016/j.fitote.2012.11.011 23219978

[B90] Teng S. Wang Y. Li P. Liu J. Wei A. Wang H. (2017). Effects of R type and S type ginsenoside Rg3 on DNA methylation in human hepatocarcinoma cells. Mol. Med. Rep. 15 (4), 2029–2038. 10.3892/mmr.2017.6255 28260016 PMC5364960

[B91] Trinh H. T. Han S. J. Kim S. W. Lee Y. C. Kim D. H. (2007). Bifidus fermentation increases hypolipidemic and hypoglycemic effects of red ginseng. J. Microbiol. Biotechnol. 17 (7), 1127–1133. 18051323

[B92] Wang W. Zhao Y. Rayburn E. R. Hill D. L. Wang H. Zhang R. (2007). *In vitro* anti-cancer activity and structure-activity relationships of natural products isolated from fruits of Panax ginseng. Cancer Chemother. Pharmacol. 59 (5), 589–601. 10.1007/s00280-006-0300-z 16924497

[B93] Wei W. Liu L. Liu X. Tao Y. Gong J. Wang Y. (2022). Black ginseng protects against Western diet-induced nonalcoholic steatohepatitis by modulating the TLR4/NF-κB signaling pathway in mice. J. Food Biochem. 46 (12), e14432. 10.1111/jfbc.14432 36183169

[B94] Wei X. Chen J. Su F. Su X. Hu T. Hu S. (2012). Stereospecificity of ginsenoside Rg3 in promotion of the immune response to ovalbumin in mice. Int. Immunol. 24 (7), 465–471. 10.1093/intimm/dxs043 22427454

[B95] Wei Y. Zhao W. Zhang Q. Zhao Y. Zhang Y. (2011). Purification and characterization of a novel and unique ginsenoside Rg1-hydrolyzing β-d-Glucosidase from Penicillium sclerotiorum. Acta biochimica biophysica Sinica 43 (3), 226–231. 10.1093/abbs/gmr001 21297118

[B96] Xie Y. Y. Luo D. Cheng Y. J. Ma J. F. Wang Y. M. Liang Q. l. (2012). Steaming-induced chemical transformations and holistic quality assessment of red ginseng derived from Panax ginseng by means of HPLC-ESI-MS/MS(n)-based multicomponent quantification fingerprint. J. Agric. Food Chem. 60 (33), 8213–8224. 10.1021/jf301116x 22839102

[B97] Xiong Y. Halima M. Che X. Zhang Y. Schaaf M. J. M. Li M. (2022). Steamed Panax notoginseng and its saponins inhibit the migration and induce the apoptosis of neutrophils in a zebrafish tail-fin amputation model. Front. Pharmacol. 13, 946900. 10.3389/fphar.2022.946900 35873541 PMC9302486

[B98] Xu F. L. Zhang Q. Y. Jiang L. Peng G. M. Yao R. Li B. T. (2016). Study on chemical constituents of radix ginseng destillata alcohol extract by UHPLC-Q-TOF/MS. Traditional Chin. Drug Res. Clin. Pharmacol. 26 (4), 529–534.

[B99] Xu X. F. Gao Y. Xu S. Y. Liu H. Xue X. Zhang Y. (2018). Remarkable impact of steam temperature on ginsenosides transformation from fresh ginseng to red ginseng. J. Ginseng Res. 42 (3), 277–287. 10.1016/j.jgr.2017.02.003 29983609 PMC6026370

[B100] Xu X. F. Nie L. X. Pan L. L. Hao B. Yuan S. X. Lin R. C. (2014). Quantitative analysis of Panax ginseng by FT-NIR spectroscopy. J. Anal. Methods Chem. 2014 (8), 741571. 10.1155/2014/741571 24883224 PMC4026986

[B101] Yamabe N. Kim Y. J. Lee S. Cho E. J. Park S. H. Ham J. (2013). Increase in antioxidant and anticancer effects of ginsenoside Re-lysine mixture by Maillard reaction. Food Chem. 138 (2-3), 876–883. 10.1016/j.foodchem.2012.12.004 23411191

[B102] Yang H. Oh K. H. Kim H. J. Cho Y. H. Yoo Y. C. (2018). Ginsenoside-Rb2 and 20(S)-Ginsenoside-Rg3 from Korean red ginseng prevent rotavirus infection in newborn mice. J. Microbiol. Biotechnol. 28 (3), 391–396. 10.4014/jmb.1801.01006 29316736

[B103] Yoo S. Park B. I. Kim D. H. Lee S. Lee S. H. Shim W. S. (2021). Ginsenoside absorption rate and extent enhancement of black ginseng (CJ EnerG) over red ginseng in healthy adults. Pharmaceutics 13 (4), 487. 10.3390/pharmaceutics13040487 33918329 PMC8067055

[B104] Zhang H. M. Li S. L. Zhang H. Wang Y. Zhao Z. L. Chen S. L. (2012). Holistic quality evaluation of commercial white and red ginseng using a UPLC-QTOF-MS/MS-based metabolomics approach. J. Pharm. Biomed. Anal. 62, 258–273. 10.1016/j.jpba.2012.01.010 22310552

[B105] Zhang Z. Zhang Y. Gao M. Cui X. Yang Y. van Duijn B. (2019). Steamed Panax notoginseng attenuates anemia in mice with blood deficiency syndrome via regulating hematopoietic factors and JAK-STAT pathway. Front. Pharmacol. 10, 1578. 10.3389/fphar.2019.01578 32038252 PMC6985777

[B106] Zheng Q. Li Z. Liu J. Han L. Zhang N. Zhang H. (2016). Two new dammarane-type triterpene sapogenins from Chinese red ginseng. Nat. Prod. Res. 30 (1), 95–99. 10.1080/14786419.2015.1038538 26156746

[B107] Zhou Q. L. Xu W. Yang X. W. (2016). Chemical constituents of Chinese red ginseng. Zhongguo Zhong Yao Za Zhi 41 (2), 233–249. 10.4268/cjcmm20160214 28861969

[B108] Zhou Q. L. Yang X. W. (2015). Four new ginsenosides from red ginseng with inhibitory activity on melanogenesis in melanoma cells. Bioorg Med. Chem. Lett. 25 (16), 3112–3116. 10.1016/j.bmcl.2015.06.017 26087936

[B109] Zhu L. Luan X. Dou D. Huang L. (2019). Comparative analysis of ginsenosides and oligosaccharides in white ginseng (WG), red ginseng (RG) and black ginseng (BG). J. Chromatogr. Sci. 57 (5), 403–410. 10.1093/chromsci/bmz004 30839052

